# Analysis of biofilm complexity in 3D: an open-source framework for quantitative fractal, textural and statistical analysis of colony biofilm morphology in three dimensions

**DOI:** 10.1098/rsos.260853

**Published:** 2026-11-11

**Authors:** Gail McConnell

**Affiliations:** Strathclyde Institute of Pharmacy and Biomedical Sciences, University of Strathclyde, Glasgow, UK

**Keywords:** biofilm, microscopy, image analysis, fluorescence

## Abstract

Quantitative image analysis is central to understanding microbial growth, morphology and spatial organization. However, conventional metrics such as mean intensity or object count often do not capture the complex structural heterogeneity and patterning characteristic of microbial colonies and biofilms. To address this limitation, analysis of biofilm complexity in 3D (ABC3D), an open-source Python framework for automated extraction of fractal, textural and statistical descriptors from volumetric microscopy images, is reported. ABC3D computes a set of parameters including fractal dimension, lacunarity, entropy, grey-level co-occurrence matrix features and relative wavelet sub-band energies from three-dimensional image datasets. ABC3D is demonstrated in *Escherichia coli* macrocolony biofilms, where the first two principal components captured 75.5% of architectural variance. Nutrient environment, genetic background and their interaction significantly influenced biofilm architecture, indicating that structural responses to nutrient conditions are strain-dependent. Analysis of individual parameters revealed coordinated changes in fractal, textural and spatial-frequency features rather than uniform changes in a single measure of complexity. ABC3D provides researchers with an accessible, quantitative approach to assessing biofilm morphology in microscopy datasets.

## 1. Introduction

Understanding how biofilms are structured is essential for interpreting how they function and respond to environmental stress. Confocal laser scanning microscopy (CLSM) has made high-resolution imaging of biofilms possible in two dimensions and three dimensions [1,2], but the field has been slow to develop the software tools necessary for measurement of these complex living structures. Early tools such as COMSTAT provided fundamental but important measurements from CLSM data such as biofilm volume and surface-to-volume ratio, and these enabled comparisons of biofilm architecture in different environmental conditions [3]. COMSTAT, and subsequently COMSTAT2 [4], has helped to establish a solid basis for morphological measurements of biofilms.

Further image data approaches such as digital image in microbial ecology (daime) [5] and later biofilm intensity and architecture measurement [6] extended measurement capability to include segmentation, two dimensional/three dimensional object quantification and visualization for microbial communities. More recently, BiofilmQ has extended capability further, offering cell segmentation and measurement in space and time [7].

Biofilm architecture maps directly onto mass transport, reaction and detachment phenomena [8,9], and thicker biofilms have been reported to harbour deeper gradients of oxygen and nutrients, which can modulate growth [10–12]. Measurement of these properties has assisted in understanding the relationship between architecture and function [13].

Architecture also shapes antimicrobial tolerance. The extracellular matrix (extracellular polymeric substances (EPS)) can impede diffusion and create chemical microenvironments that inhibit growth and change antibiotic susceptibility [14]. Increases in thickness, roughness and porosity often correlate with reduced penetration and elevated tolerance [13].

Beyond gross morphology, texture metrics, including fractal dimension, lacunarity, grey-level co-occurrence matrix (GLCM) statistics, entropy and multi-scale spectral measures, capture heterogeneity and self-similarity across scales [15–18] and are distinct from volume and thickness. This is shown schematically in figure 1. Fractal dimension quantifies the complexity and space-filling organization of biomass across scales, while lacunarity captures the distribution and size of gaps and is therefore informative about biofilm porosity and channel organization. Entropy and GLCM statistics describe complementary aspects of spatial heterogeneity and local organization, and relative wavelet sub-band energies indicate how structural variation is distributed across spatial scales and directions. Together, these descriptors distinguish dense, homogeneous architectures from heterogeneous, channel-rich structures. They should, however, be interpreted as quantitative measures of biofilm architecture rather than as direct or uniquely specific readouts of individual biological processes.

**Figure 1.**
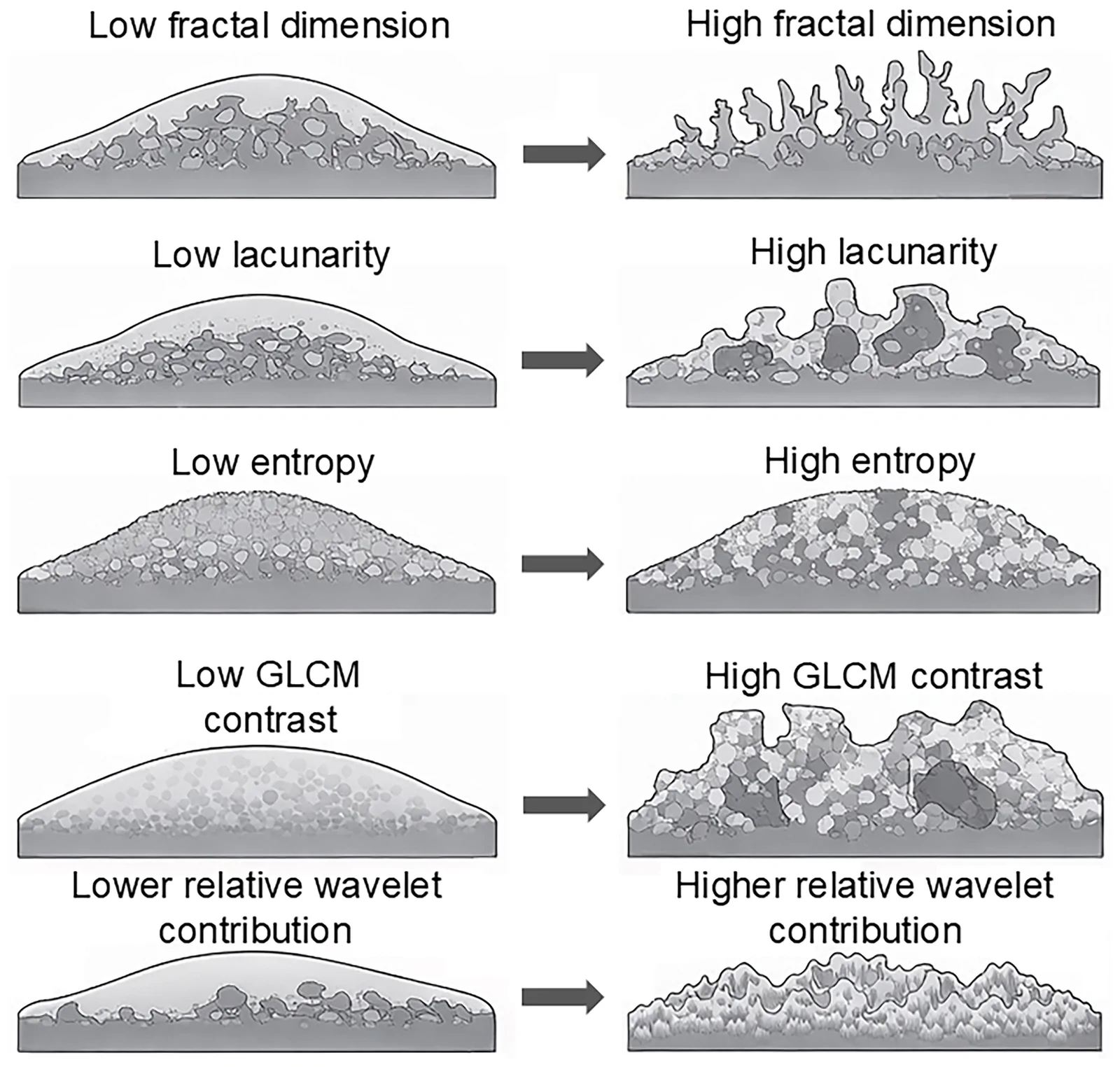
Schematic illustration of multi-scale architectural descriptors computed by ABC3D. Schematic cross-sectional (xz) representations of biofilm structure demonstrating how key quantitative descriptors vary from low (left) to high (right) values. Increasing fractal dimension reflects greater scale-invariant structural complexity and space-filling branching. Increasing lacunarity indicates greater heterogeneity in gap size and spatial distribution of voids. Increasing entropy represents greater voxel-level intensity heterogeneity within the biofilm volume. Increasing GLCM contrast reflects stronger local intensity differences between neighbouring pixels within the image planes and greater textural roughness. Relative wavelet sub-band energy represents the proportion of total wavelet energy contained within a given spatial-frequency and directional component. Illustrations are conceptual and intended to provide intuitive interpretation of quantitative metrics rather than report direct biological measurements.

Early work to extend fractal and texture-based analysis to three-dimensional biofilm datasets included image structure analyser in 3D (ISA3D) software, which measured the box-counting fractal dimension, lacunarity and grey-level texture of confocal image stacks [19]. ISA3D demonstrated that volumetric biofilm complexity could be quantified beyond traditional bulk parameters, and this represented an important methodological advance at the time. However, the software was distributed as MATLAB-based code and is no longer actively maintained or publicly accessible. Contemporary tools increasingly support these measurements, but workflows typically still require stitching together multiple libraries or software apps. These limitations highlight the need for a transparent technology for multi-scale three-dimensional architectural phenotyping.

Analysis of biofilm complexity in 3D (ABC3D) is introduced as an open-source Python framework that unifies a broad range of quantitative descriptors relevant to biofilm image data analysis. It consolidates fractal measures, textural features derived from GLCMs, energy parameters and statistical analyses within a single workflow. By analysing three dimensional data, ABC3D addresses limitations inherent in two-dimensional analysis. This framework provides microbiologists with a flexible and extendable tool for linking biofilm architecture and texture to underlying physiological and ecological processes.

## 2. Material and methods

A schematic showing the experimental and computational pipeline is shown in figure 2.

**Figure 2.**
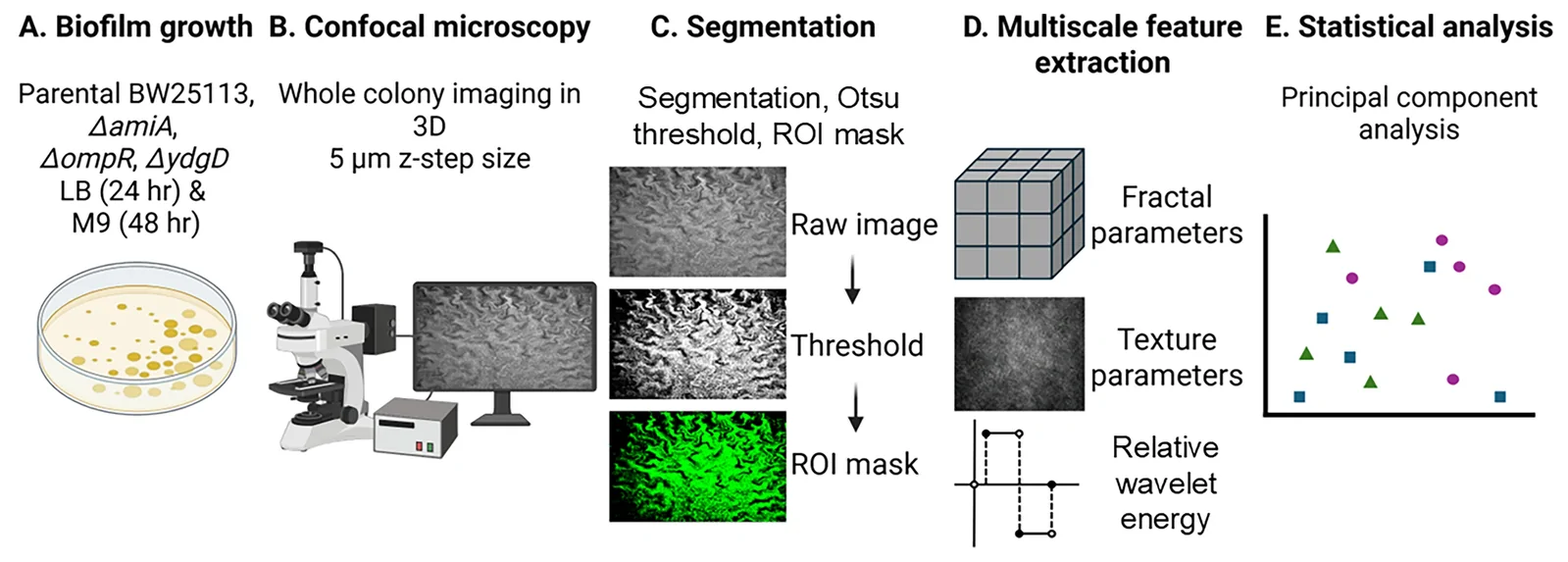
Experimental and computational workflow for multi-scale biofilm architecture analysis. (A) Biofilm growth conditions. Parental (BW25113) and deletion mutants (*ΔamiA*, *ΔompR* and *ΔydgD*) were grown as single-founder, isogenic macrocolony biofilms on lysogeny broth (LB) agar (24 hours) and M9 minimal agar (48 hours). (B) Whole colony three-dimensional image stacks were acquired by confocal laser scanning microscopy using a 5 μm z-step size to capture full colony architecture. (C) Raw volumetric images were segmented using Otsu thresholding to generate a binary region-of-interest (ROI) mask representing biofilm biomass. (D) Quantitative descriptors were computed from segmented volumes, including fractal parameters, texture features derived from GLCMs and relative wavelet sub-band energy metrics. (E) Feature matrices were standardized, and principal component analysis (PCA) was performed to identify dominant axes of structural variation, followed by factorial and permutation-based statistical analyses to assess the effects of strain and growth medium on biofilm architecture.

### 2.1. Software architecture and computation of features

ABC3D is a standalone, script-based workflow written in Python (v. 3.11.13). The analysis was implemented as custom Python code using libraries from the scientific Python ecosystem, including NumPy, pandas, tifffile, scikit-image, SciPy and PyWavelets. Code development and execution were performed in the Spyder (v. 6.0.8) integrated development environment, and the software was run on an HP EliteBook 830 laptop computer with an Intel® Core™ Ultra 5 1.6 GHz processor and 16 GB RAM, with a Microsoft Windows 11 Enterprise operating system.

Three-dimensional OME-TIFF stacks are imported and standardized to (z, y, x) ordering. Intensity data are converted to 32-bit floating point format to ensure numerical stability and memory efficiency. Biofilm biomass is segmented from background using global intensity thresholding. Otsu's method is applied to a reproducible random subsample of up to 10⁶ voxels rather than the full volume [20]. This sampling limit is set to control processing time, rather than being a memory constraint. Optional parameters allow manual threshold specification and removal of small, connected components below a user-defined voxel size to suppress noise. The resulting binary mask defines the biofilm region of interest (ROI) for all subsequent analyses.

Structural quantification was performed directly on the segmented three-dimensional volume. Total foreground voxel count and foreground fraction were calculated to estimate biomass occupancy within the imaged volume. Intensity heterogeneity within the volume was quantified using Shannon entropy and Rényi entropy (*α* = 2) [15,21].

Scale-dependent architectural complexity was assessed using fully three-dimensional box-counting analysis and lacunarity. Fractal dimension was obtained via relative dimension box-counting (RDBC) from log–log regression across dyadic box sizes, with *R*² reported as a goodness-of-fit metric [22]. Lacunarity was computed across equivalent spatial scales to characterize structural heterogeneity and gap distribution within the biofilm [17].

Local spatial organization was evaluated using slice-wise masked GLCMs (2.5D GLCM), from which Haralick statistics (angular second moment, contrast, correlation, dissimilarity, energy and homogeneity) were summarized across slices [16].

Multi-scale structural organization was further characterized using a single-level three-dimensional discrete wavelet transform (Daubechies db2). Following mean-centring of intensity values within the biofilm ROI, energy was calculated for each of the eight three-dimensional sub-bands (LLL, LLH, LHL, LHH, HLL, HLH, HHL and HHH), where H = high and L = low. To reduce dependence on overall signal magnitude and ROI size, each sub-band energy was expressed relative to the total wavelet energy across all eight sub-bands. These relative energies therefore describe the distribution of image variation across spatial-frequency and directional components rather than absolute image energy [18,23,24].

All computed features were exported as a single .CSV file. All analysis parameters were explicitly defined within the script to ensure full reproducibility. Full mathematical definitions of all computed descriptors are provided in the supplementary material.

### 2.2. Experimental design and image data acquisition

No new image data were acquired for demonstrating ABC3D. Instead, data were repurposed from Bottura *et al.* [25], where full information on specimen preparation is available. A brief overview of specimen preparation and image data acquisition is presented here.

The bacterial strains used were obtained from the Keio collection [26], a single-gene knockout library of all non-essential genes in the *Escherichia coli* K-12 strain, BW25113 [27]. Green fluorescent protein (GFP)-expressing strains, parental BW25113/pAJR145, BW25113 *ΔamiA*::kan/pAJR145, BW25113 *ΔompR*::kan/pAJR145 and BW25113 *ΔydgD*::kan/pAJR145 (referred to here as *ΔamiA*, *ΔompR* and *ΔydgD*), were selected for their modified cell phenotype. Macrocolony biofilms of each strain were grown on agar substrates in a sterile 3D-printed plastic specimen holder, as described previously [28]. Macrocolony biofilms were grown for 24 hours when using Miller's lysogeny broth (LB) and for 48 hours when using M9 minimal medium supplemented with 0.2% glucose. The longer incubation period in M9 reflected the slower growth of *E. coli* in minimal medium compared with LB [29].

Mature colony biofilm images were acquired on an Olympus IX81 microscope coupled to a FluoView FV1000 confocal laser scanning unit (Olympus, Japan). Fluorescence from GFP was excited using a 488 nm argon laser (GLG3135, Showa Optronics, Japan) and was detected by a photomultiplier tube with a spectral detection window set between wavelengths of 510 and 560 nm. Samples were imaged using a 10×/0.4 dry objective lens.

Three-dimensional z-stacks of colony biofilms grown on LB and M9 media were acquired with a slice spacing of 5 µm. Colony biofilms were imaged for each strain and each medium (*n* = 5 repeats). All data were saved in the proprietary .oib file format and were converted to OME.TIFF in FIJI [30]. Representative maximum intensity projection images, also made in FIJI, of each strain and nutrient are shown in figure 3.

**Figure 3.**
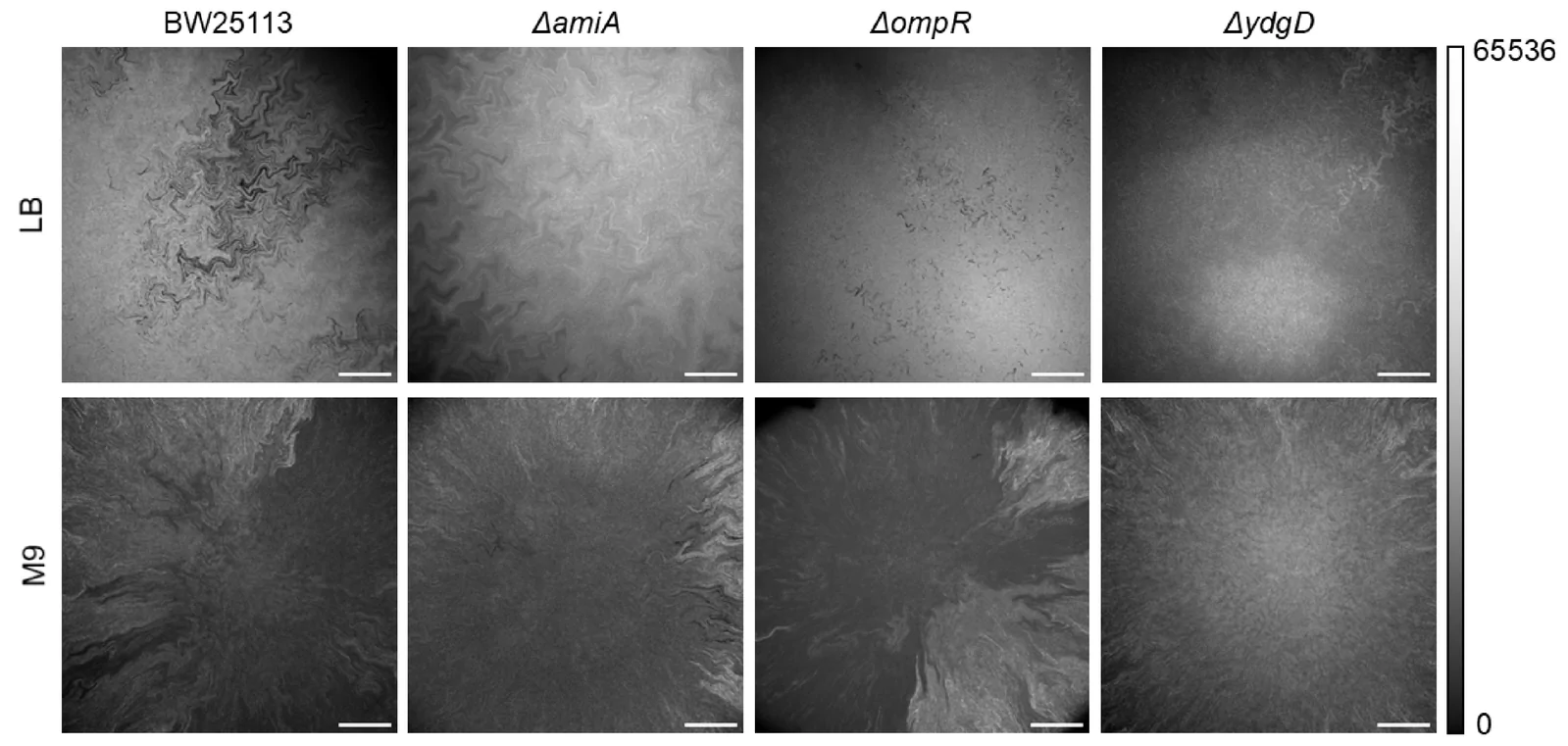
Representative maximum intensity projection images of confocal microscopy datasets showing the morphology of biofilms formed by the strains BW25113 (parental), *ΔamiA*, *ΔompR*, and *ΔydgD* in LB and M9 media. Scale bar = 200 µm.

### 2.3. Statistical analysis

Statistical analyses of all image datasets were performed in Python using scikit-learn [31] and statsmodels [32]. Quantitative features derived from ABC3D, including entropy measures, fractal dimension, lacunarity, GLCM statistics and relative wavelet sub-band energies, were assembled into a single data matrix in which each row represented one imaged biofilm and each column represented one measured parameter. GLCM energy was excluded from the multivariate analysis because it is mathematically dependent on the angular second moment. This matrix formed the basis for subsequent multivariate statistical analysis.

#### 2.3.1. Principal component analysis

Principal component analysis (PCA) was used to simplify the dataset by summarizing multiple architectural measurements into a smaller number of composite variables [33]. Before PCA, all features were standardized using zero-mean, unit-variance scaling so that measurements with larger numerical ranges did not disproportionately influence the analysis.

PCA transforms the original correlated features into new, uncorrelated variables called principal components (PCs). Each PC represents a distinct pattern of variation in biofilm architecture. For each biofilm, a PC score indicates its position along a given component axis, effectively summarizing how strongly that biofilm expresses the structural behaviour described by that component.

Each component explains a proportion of the total variance in the dataset. Components are ordered from highest to lowest explained variance, meaning that the first component (PC1) captures the largest share of overall architectural variation across biofilms, the second component (PC2) captures the next largest share and so on. The first two components (PC1 and PC2) were retained for visualization and subsequent statistical analysis, as together they accounted for most of the total variance.

Feature loadings were examined to identify which architectural measurements contributed most strongly to variation along each component axis.

#### 2.3.2. Two-way ANOVA on principal components

To assess the contributions of genetic background and growth medium to biofilm architecture, two-way ANOVA models were fitted to PC1 and PC2 scores using ordinary least squares regression with strain and medium as fixed factors, including a strain × medium interaction term. Type II sums of squares were used for the factorial models, with main effects interpreted alongside the interaction term. All tests were two-sided, and statistical significance was defined as *P* < 0.05.

#### 2.3.3. Permutation-based multivariate analysis

To test whether the experimental factors explained multivariate architectural variation beyond that expected under a null model, permutation-based multivariate analysis was performed on the complete standardized feature matrix used for PCA. Growth medium, strain and their interaction were evaluated using partial pseudo-*F* statistics in a factorial multivariate regression framework. Statistical significance was assessed using Freedman–Lane residual permutation with 9999 random permutations. For each term, residuals from the corresponding reduced model were randomly permuted and added to the fitted values before refitting the full and reduced models. The permutation *P*-value was calculated as the proportion of permuted pseudo-*F* statistics equal to or greater than the observed statistic, with the standard +1 correction. For each experimental term, an effect-size measure was calculated as the partial term sum of squares, obtained from the difference in residual sum of squares between the reduced and full models, divided by the total sum of squares of the standardized multivariate feature matrix. This quantity represents the proportion of total multivariate variance uniquely attributable to that term conditional on the remaining terms in the model. PCA was used for unsupervised visualization and was not used as a statistical test of group separation.

#### 2.3.4. Data presentation

PCA scatter plots, interaction plots (showing group means ± standard error of the mean, s.e.m.) and heat maps of mean *z*-scored feature values grouped by strain × medium were generated from ABC3D outputs using custom Python code. Permutation null distributions and the corresponding observed pseudo-*F* statistics were calculated using a separate custom Python script.

ANOVA results are reported as *F*(*df*_1_,*df*_2_) = value, *P* = value, where *df*_1_ and *df*_2_ denote numerator and denominator degrees of freedom, respectively. All statistical tests were two-sided unless otherwise stated.

## 3. Results

### 3.1. Multi-scale feature extraction with analysis of biofilm complexity in three dimensions captures dominant axes of architectural variation

Biofilm architecture was measured across genetic backgrounds and nutrient conditions by extracting structural features from three-dimensional confocal stacks using ABC3D.

PCA of the multivariate architectural feature set revealed that PC1 accounted for 49.1% of total variance and PC2 accounted for 26.4%, together representing 75.5% of the measured architectural variation across all samples (figure 4). Visualization and interpretation therefore focused on PC1-PC2 space. PCA was used to describe the dominant axes of variation in the multivariate architectural dataset rather than as a statistical test of experimental group separation.

**Figure 4.**
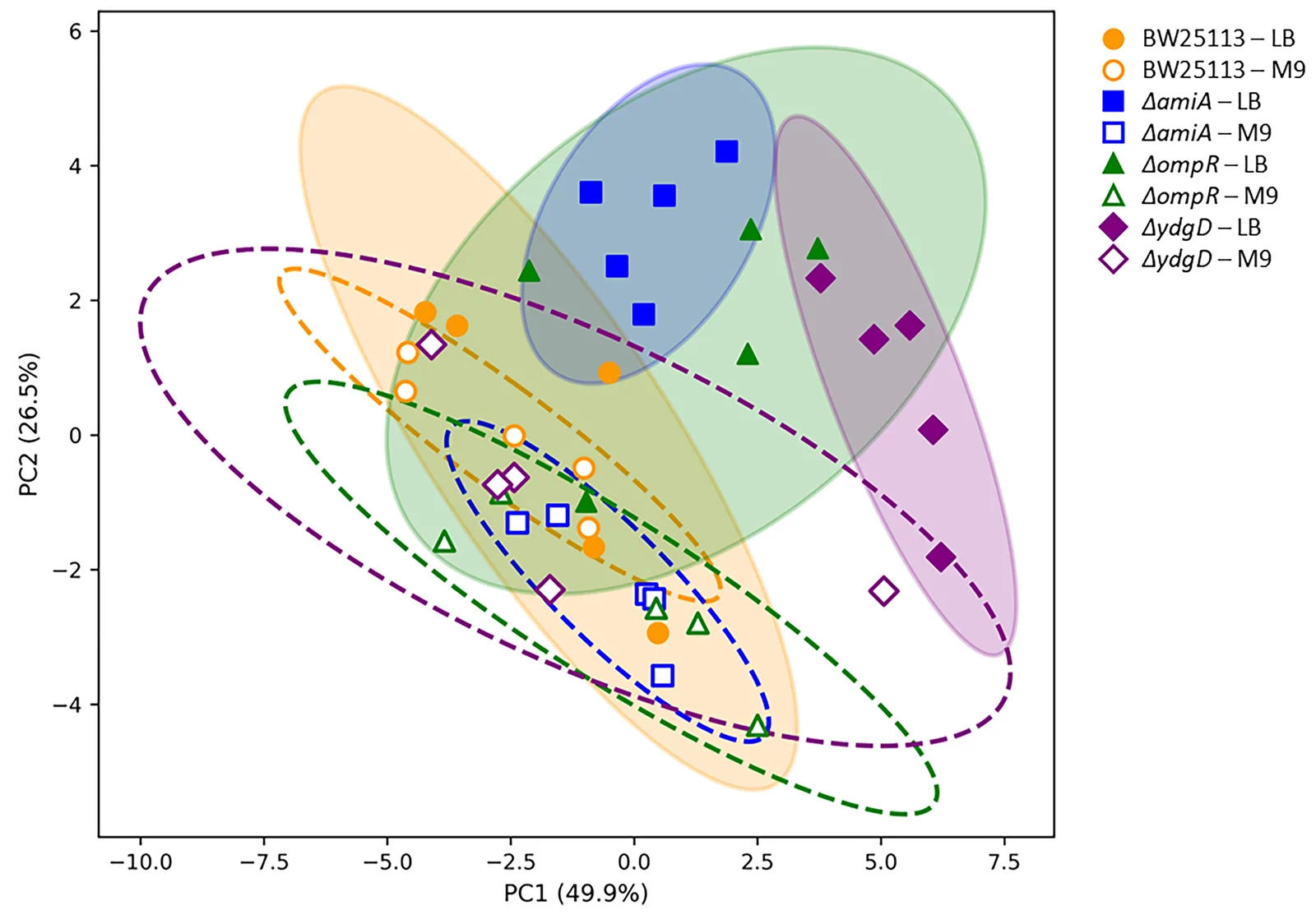
PCA of standardized biofilm architectural descriptors derived from *n* = 5 independent three-dimensional confocal image stacks per strain × medium condition using ABC3D. PC1 and PC2 account for 49.1 and 26.4% of total variance, respectively. PCA provides an unsupervised visualization of the dominant axes of architectural variation; statistical evidence for effects of strain, growth medium and their interaction was assessed independently using factorial and permutation-based analyses. Ellipses represent covariance-based data ellipses (2 s.d.) calculated from PC1 and PC2 scores for each strain × medium group and illustrate within-group dispersion rather than statistical confidence regions.

### 3.2. Growth medium and genetic background jointly influence biofilm architecture

Projection of samples into PC space revealed structured variation associated with growth medium and strain, although substantial overlap was evident between several strain × medium groups (figure 4). PCA was therefore treated as a descriptive representation of the dominant axes of architectural variation rather than as evidence of discrete group separation.

Two-way ANOVA of the PC scores demonstrated significant effects on PC1 of growth medium (*F*_1,32_ = 7.94, *P* = 0.0082), strain (*F*_3,32_ = 6.14, *P* = 0.0020) and the strain × medium interaction (*F*_3,32_ = 3.80, *P* = 0.0194). The interaction plot illustrates that the effect of growth medium on PC1 differed among strains (figure 5A). *ΔydgD* showed the largest shift, with substantially higher PC1 scores in LB than in M9, whereas *ΔamiA* showed comparatively little change between media. BW25113 and *ΔompR* showed smaller medium-dependent changes.

**Figure 5.**
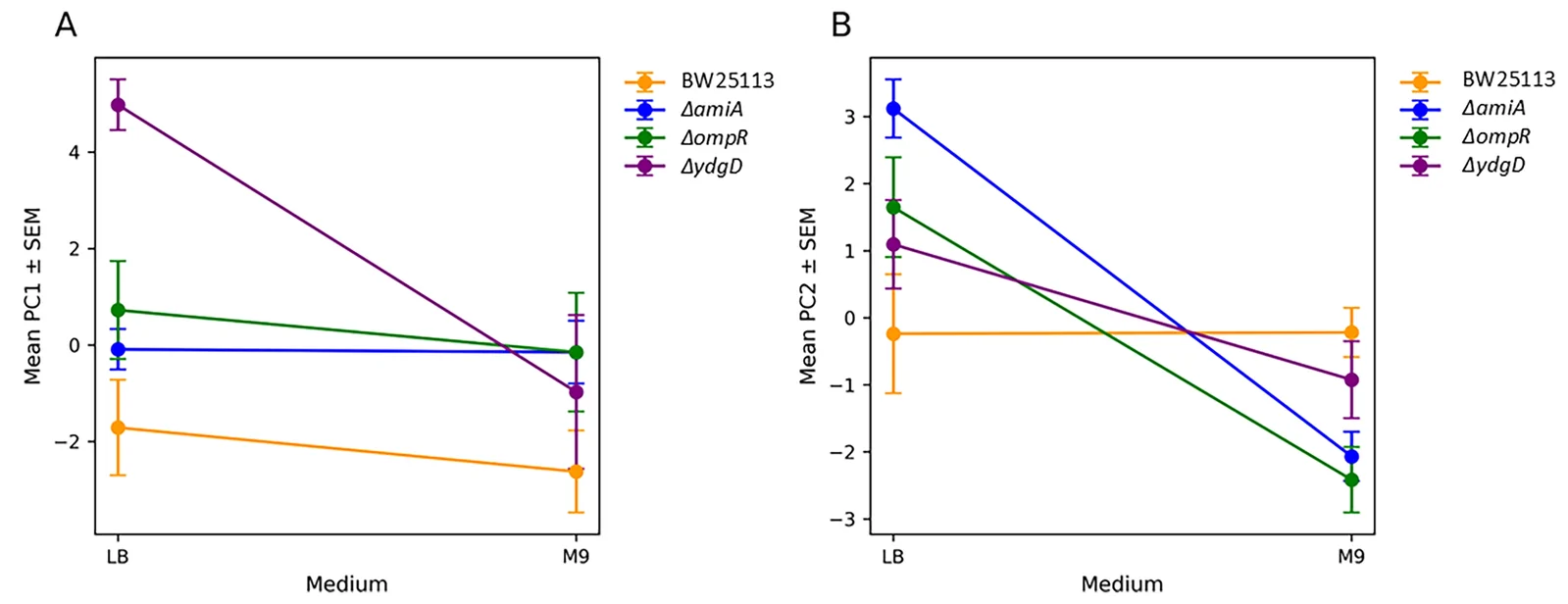
(A) Mean PC1 scores (± s.e.m.) and (B) mean PC2 scores (± s.e.m.) for each strain grown in LB or M9 medium (*n* = 5 independent biofilms per condition). Non-parallel trajectories illustrate that the magnitude of the response to growth medium differs among strains. Statistical effects were assessed using two-way ANOVA; multivariate effects across the complete feature matrix were assessed independently using permutation-based analysis.

For PC2, there was a strong effect of growth medium (*F*_1,32_ = 45.15, *P* < 0.0001) and a significant strain × medium interaction (*F*_3,32_ = 7.54, *P* = 0.00060), whereas the strain main effect was not significant (*F*_3,32_ = 0.92, *P* = 0.443). Here, *ΔamiA* and *ΔompR* showed pronounced decreases in PC2 score from LB to M9, while *ΔydgD* showed a smaller decrease and BW25113 changed comparatively little (figure 5B). Thus, the absence of an overall strain effect on PC2 does not indicate a uniform response across strains; rather, the significant interaction reflects differences among strains in their response to growth medium.

To determine whether these experimental effects were also detectable in the complete multivariate feature space, independently of visual separation in the PCA projection, permutation-based multivariate analysis was performed on the standardized feature matrix. These data are shown in figure S1 in the supplementary material. Growth medium accounted for 18.9% of the total variance in the standardized multivariate feature matrix (pseudo-*F* = 13.309, permutation *P* = 0.0001), strain accounted for 17.4% (pseudo-*F* = 4.087, *P* = 0.0009) and the strain × medium interaction accounted for 18.3% (pseudo-*F* = 4.292, *P* = 0.0006). In each case, the observed pseudo-*F* statistic lay in the extreme upper tail of its corresponding permutation null distribution, demonstrating that the association between the experimental factors and multivariate architectural variation was greater than expected under the corresponding null model.

Together, these analyses provide evidence that the measured biofilm architectural descriptors are influenced by both growth medium and strain, with a significant interaction demonstrating that the architectural response to nutrient environment depends upon genetic background.

### 3.3. Principal component 1 captures a texture homogeneity-contrast axis

The largest absolute loadings for PC1 are shown in figure 6A. Positive contributions were dominated by GLCM homogeneity, LLL relative wavelet energy, GLCM angular second moment and RDBC, whereas negative contributions included GLCM dissimilarity, contrast and several relative wavelet-energy components. PC1 therefore broadly represents a continuum between locally smoother, more homogeneous architectures and structures exhibiting greater local contrast and higher-frequency spatial variation. Following normalization of the wavelet features, PC1 was no longer dominated by absolute wavelet-energy magnitude, indicating that this axis reflects the relative distribution of architectural information across complementary textural and spatial-frequency descriptors.

**Figure 6.**
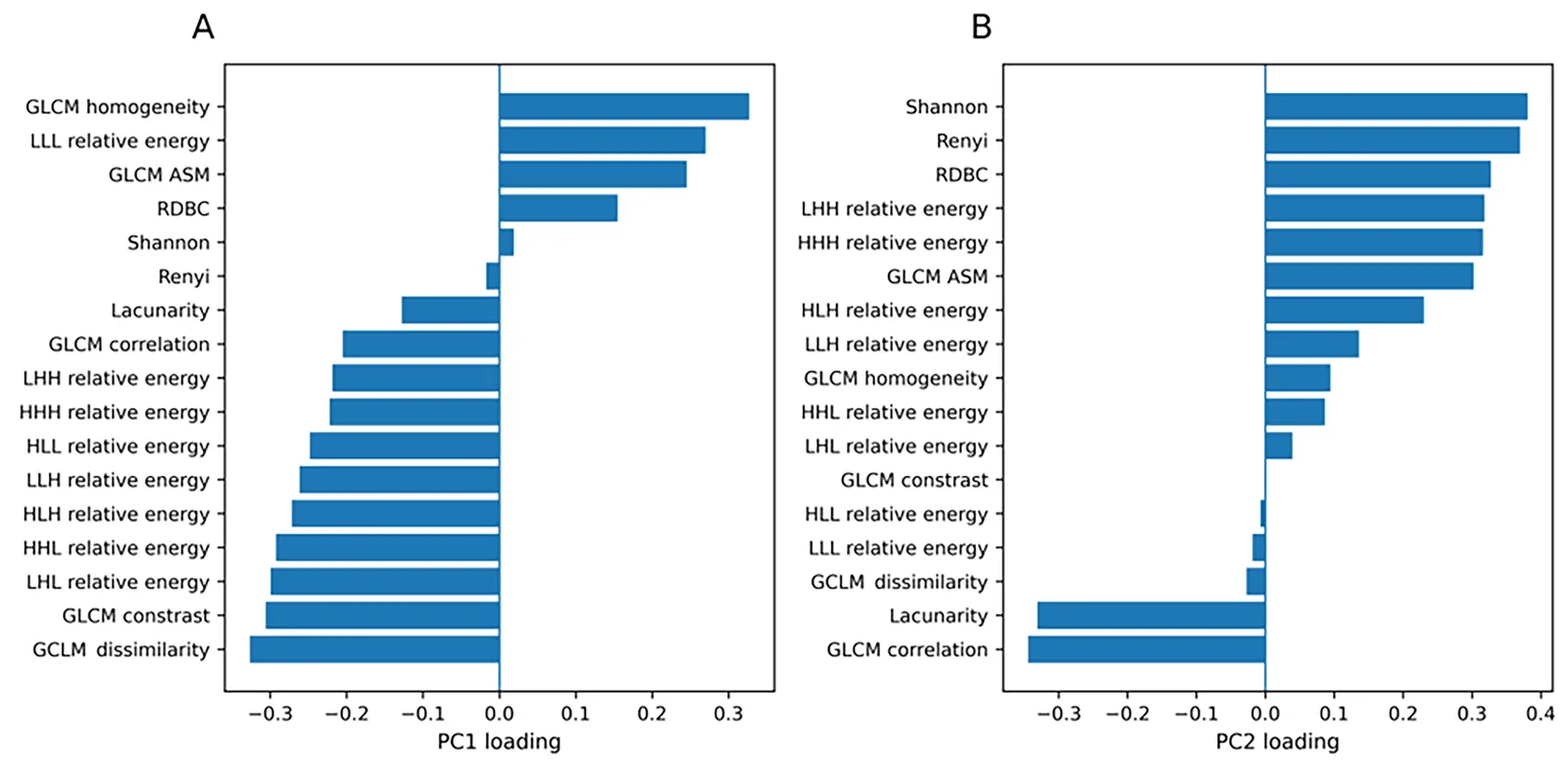
Principal component loadings reveal complementary features associated with biofilm architectural variation. (A) PC1 loadings show positive contributions from GLCM homogeneity, LLL relative wavelet energy, GLCM angular second moment and RDBC, opposed by GLCM dissimilarity, contrast and several relative wavelet-energy components. (B) PC2 is characterized by positive contributions from Shannon and Rényi entropy, RDBC, GLCM angular second moment and selected relative wavelet-energy components, with negative contributions from GLCM correlation and lacunarity. Loadings indicate the contribution and direction of individual descriptors to each principal component.

### 3.4. Principal component 2 captures complementary structural heterogeneity

PC2 describes a complementary form of architectural variation (figure 6B). Positive loadings included Shannon and Rényi entropy, RDBC, GLCM angular second moment and selected relative wavelet energy components, whereas GLCM correlation and lacunarity contributed negatively. PC2 therefore reflects coordinated variation in intensity heterogeneity, fractal organization and spatial-frequency composition rather than a single structural property.

### 3.5. Architecture varies across genetic backgrounds and growth environments

The mean *z*-score heat map by strain × medium (figure 7) further demonstrated that architectural differences reflected coordinated changes across multiple descriptor families rather than uniform changes in a single measure. *ΔydgD* grown in LB exhibited a particularly distinctive profile, characterized by higher GLCM homogeneity and angular second moment together with lower contrast, dissimilarity and several relative wavelet-energy components. *ΔamiA* grown in LB showed comparatively higher entropy and selected higher-frequency relative wavelet energies, whereas *ΔompR* grown in M9 exhibited lower Shannon and Rényi entropy. These patterns demonstrate that the measured architectural response to growth medium differs among genetic backgrounds.

**Figure 7.**
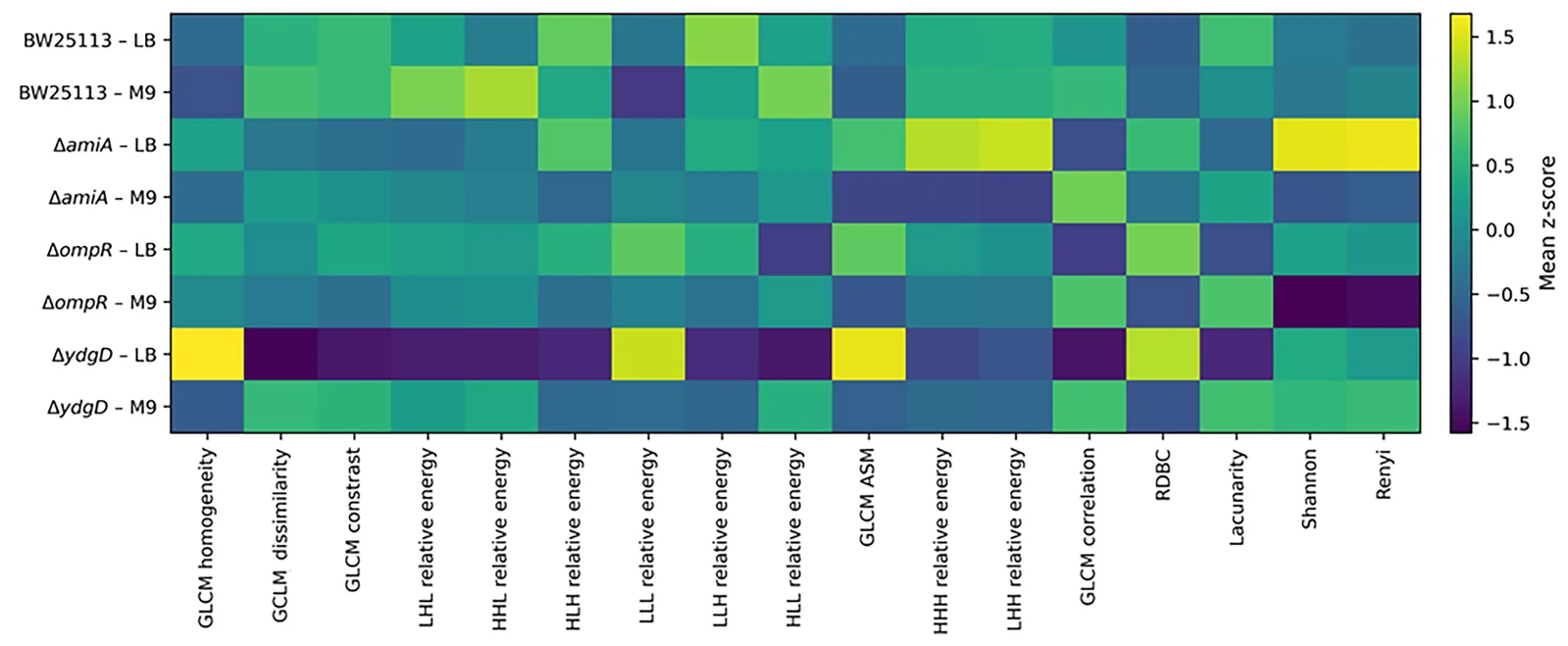
Mean standardized architectural feature profiles across strain × medium conditions. Rows represent experimental groups and columns represent structural descriptors, including relative wavelet sub-band energies, GLCM texture metrics, RDBC, lacunarity and entropy measures. Values are mean *z*-scores for *n* = 5 independent biofilms per condition. The heat map demonstrates condition-dependent redistribution across multiple descriptor families rather than uniform changes in architectural complexity, with distinct responses evident among strains and growth media.

The observed shifts were not monotonic across all descriptors, indicating that architectural changes do not correspond to a uniform increase or decrease in complexity. Instead, the different strain × medium conditions are characterized by redistribution across fractal, textural and spectral features.

## 4. Discussion

ABC3D is presented as a framework for defining macrocolony biofilm architecture as an integrated multi-scale phenotype rather than as a collection of independent structural descriptors. By unifying fractal geometry, lacunarity, entropy, grey-level texture statistics and three-dimensional relative wavelet sub-band energy features within a single reproducible workflow, ABC3D resolves architectural organization as a coordinated spatial system. In doing so, it moves beyond conventional measures of biomass, thickness or single-parameter complexity and instead maps biofilms within a multidimensional architectural space.

Parameters such as fractal dimension have previously been studied in *E. coli* biofilms [25], and texture analysis has been performed in biofilms formed by *Pseudomonas aeruginosa, Pseudomonas fluorescens* and *Klebsiella pneumoniae* [34]. However, these measurements were made from two-dimensional projections and therefore do not fully capture the volumetric organization of intact biofilms.

The datasets analysed here were previously assessed using two-dimensional RDBC analysis of maximum intensity projections to quantify intra-colony channel fractal complexity [25]. That study reported high fractal complexity across phenotypes and growth conditions and identified *ΔompR* as exhibiting reduced fractal dimension when grown in M9 relative to LB. Consistent with those findings, the present three-dimensional analysis also identifies a nutrient-dependent architectural response in *ΔompR*. However, the multivariate analysis demonstrates that this response is not restricted to fractal dimension and occurs alongside coordinated changes in textural and spatial-frequency descriptors. Because maximum intensity projections compress three-dimensional structure into a single plane, depth-dependent architectural organization may not have been fully resolved in the earlier analysis.

In the present study, ABC3D was applied to the same datasets for analysis in three dimensions. The results indicate that the previously reported nutrient sensitivity of *ΔompR* is not restricted to a change in fractal magnitude. The three-dimensional analysis identifies accompanying changes in texture, entropy and the distribution of relative wavelet energy, demonstrating that nutrient conditions can alter several complementary aspects of architectural organization.

The permutation analysis indicates substantial and broadly comparable contributions from growth medium, strain and their interaction to multivariate architectural variation. The significant interaction is particularly informative because it demonstrates that the architectural effect of nutrient environment depends upon genetic background. Structural phenotypes are therefore conditional rather than fixed characteristics of individual strains. The same genetic background can occupy different multi-scale structural configurations depending upon nutrient availability [35].

This coordinated multi-scale behaviour has functional implications. Gap distribution, spatial homogeneity and textural contrast influence diffusion dynamics, metabolic stratification and mechanical stability in biofilms across species [8,10,36]. When these properties shift together rather than independently, the physical microenvironment experienced by cells is altered in a structured manner. Although antimicrobial susceptibility was not directly measured here, coordinated changes in spatial-frequency composition and heterogeneity provide a possible structural basis for variation in stress tolerance and treatment response. Architectural state may therefore contribute to emergent phenotypes such as antimicrobial resistance through its effects on penetration, nutrient gradients and local stress buffering [37].

Beyond clinical and ecological contexts, quantitative characterization of architectural reorganization may also have value in biotechnology. Symmetry breaking within biofilms can generate spatially segregated subpopulations that divide labour to cooperatively synthesize complex metabolites that are difficult or costly to produce. A framework capable of quantifying coordinated multi-scale structural shifts under different industrial conditions could therefore provide practical insight into how biofilm architecture supports or hampers biomanufacturing performance [38].

ABC3D extends existing biofilm analysis platforms by integrating analyses within a transparent, script-driven multivariate framework. The combination of unsupervised PCA, factorial analysis of principal component scores and permutation-based analysis of the complete multivariate feature matrix provides complementary descriptive and inferential assessment of architectural variation. Importantly, the significant effects of medium, strain and their interaction in the permutation analysis demonstrate that the observed associations with experimental condition are not dependent on visual interpretation of group separation in PCA space.

Several limitations of ABC3D should be acknowledged. As with any quantitative analysis of volumetric microscopy data, ABC3D measurements depend on the characteristics of the input images. Conventional confocal microscopy inherently has poorer axial than lateral spatial resolution, and volumetric datasets are also commonly acquired with anisotropic voxel dimensions, depending on the lateral pixel size and axial sampling interval. These factors should be considered when interpreting three-dimensional structural descriptors. Simple interpolation to isotropic voxel dimensions does not restore isotropic optical resolution and may itself alter image texture and spatial structure, and it is therefore not recommended as a correction for resolution anisotropy. For comparative analyses, acquisition parameters, including lateral sampling and axial step size, should therefore be kept consistent between experimental groups. Low signal-to-noise ratio, background variation and depth-dependent signal attenuation may also influence the calculated descriptors, and any preprocessing should be applied consistently across the complete dataset.

Segmentation quality should be visually assessed against the original image volume to ensure that the resulting mask appropriately represents biofilm biomass. Segmentation relied on global thresholding and may therefore not capture subtle changes in colony structure. This is particularly acute in thick macrocolony specimens where light scattering can compromise image contrast at depth. This could be improved by applying local pixel-wise thresholding methods [39]. Texture analysis was implemented slice-wise rather than as a fully volumetric operation, and only single-level wavelet sub-band decomposition was applied. Incorporation of multi-level spectral analysis and fully three-dimensional texture computation would further refine measurements.

Taken together, these findings demonstrate that biofilm architecture is best understood as a coordinated, multi-scale phenotype rather than as an aggregate of independent structural metrics. By positioning biofilms within a quantitative architectural state space, ABC3D enables systematic study of how genetic background and environment jointly influence the structural organization of colony biofilms. This framework provides a foundation for linking environmental and genetic inputs to emergent architectural properties, and for understanding how those states may subsequently shape the functional properties of biofilms.

## Supplementary Material



## Data Availability

All code is available for download at [40] and at [41]. Supplementary material is available online [42].

## Declaration of AI use

Generative AI (ChatGPT; GPT-4o and GPT-5.1; OpenAI) was used between October 2025–February 2026 to assist with debugging and annotation of analysis code. Scientific decisions, including selection of quantitative descriptors, experimental design, statistical analysis and interpretation of results, were undertaken by the author. AI-assisted code modifications were manually inspected and tested by the author, and final analyses were executed using the reviewed code against the source dataset. Numerical outputs and figures were checked for consistency with the reported results.
